# Supplementary material for: Are carbon emissions trading and green financial instruments synergistic? -Comprehensive quantitative research based on content analysis
Source: PLoS One. 2024 Mar 7;19(3):e0298601. doi: 10.1371/journal.pone.0298601 (PMC10919601; doi:10.1371/journal.pone.0298601)
Supplement: S1 Data — (ZIP) [file pone.0298601.s001.zip › Data/地级市Z_F工作报告.zip!/╣π╢1⁄2/╓╨╔╜╩╨/└┤╫╘╣1⁄2╓┌║┼í╛┬φ┐╦╩2╛▌═°í┐_2019.docx]

各位代表：

我代表市人民政府向大会作政府工作报告，请予审议，并请政协委员和其他列席人员提出意见。

2018年工作回顾

过去一年，在以习近平同志为核心的党中央坚强领导下，在省委省政府和市委的正确领导下，在市人大及其常委会和市政协的监督支持下，市政府坚持以习近平新时代中国特色社会主义思想为指导，全面贯彻党的十九大和十九届二中、三中全会精神，深入学习贯彻习近平总书记对广东重要讲话和重要指示批示精神，坚持稳中求进工作总基调，落实高质量发展要求，迎难而上、扎实工作，全市经济保持平稳健康发展，社会大局平安稳定。

（一）经济运行总体平稳。全市实现生产总值3632.7亿元，增长5.9%。人均生产总值11.1万元，位居全省前列。规上工业增加值1093.5亿元，增长3.1%。服务业增加值1790.9亿元，增长7.6%。固定资产投资1116.4亿元，增长5.5%。社会消费品零售总额1490.8亿元，增长4.5%。外贸总量扩大、结构优化，进出口总额超2300亿元，一般贸易占比达53.5%。一般公共预算收入315.2亿元，增长0.8%。完成省市重点项目投资559.2亿元。九大市级产业平台建设扎实推进，在建项目95个，总投资额544.2亿元。引进招商项目548个，总投资额1516.5亿元。

（二）质量效益稳步提升。经济结构调整持续推进，三次产业结构调整为1.7 :49 :49.3。先进制造业、高技术制造业增加值占规上工业增加值比重分别达44.7%和19.1%，现代服务业增加值占服务业增加值比重达61.8%。上规上限工程深入推进，新增规上限上企业806家，净增规上限上企业239家。新增上市公司3家。科技创新平台加快建设，中国科学院药物创新研究院中山研究院（华南分院）、哈工大无人装备与人工智能创新中心、中国科学院大学创新中心、中山—香港科技大学联合创新中心、广东药科大学—香港大学创新平台等一批科技创新平台落地，省级以上创新平台增至456家。新增国家和省级智能制造试点示范项目15个，新增国家和省级企业技术中心11家。创新主体加快发展，全市新增高新技术企业669家，总量超过2300家。国家知识产权示范城市建设扎实推进，知识产权快速维权模式在世界知识产权组织大会上得到推广。科技金融加速发展，累计发放科技贷款60亿元。科技服务业加快发展，建成技术转移和知识产权交易协同创新中心，全市技术合同交易额增长121%。创新人才加快聚集，全市共培养引进国家和省重大人才工程人才33人，国务院特殊津贴专家41人，市级以上创新创业团队40个，博士及博士后工作平台67个，新建院士工作站2个。

（三）改革开放不断深化。认真落实中央和省全面深化改革部署要求，推动115项重点领域和关键环节改革任务落地。平稳推进机构改革，政府工作机构调整为31个。深入研究贯彻落实粤港澳大湾区规划的政策措施，谋划并启动一批先期重要工作。供给侧结构性改革向纵深推进，淘汰印染行业落后产能7980万米，金融机构杠杆率保持在合理水平，降低企业成本141.8亿元，推进138个补短板项目，完成投资253.8亿元。“数字政府”建设加快推进，超过七成行政许可事项实现网上全流程办理。实施“容缺预受理”机制，推进“四减”改革，项目审批时限压缩70%以上。商事制度改革全面深化，实行营业执照“二十四证合一”，成为全国首批“企业开办全程网上办”试点城市。深化农村综合体制改革，农村土地承包经营权确权登记颁证率达95.2%。全国农村集体产权制度改革试点扎实推进，93.7%的村（社区）完成农村股份合作制改革。“网格化”综合服务管理等一批基层改革试点经验获省肯定。深化医药卫生体制综合改革，推广小榄镇医改经验，镇区医疗共同体建设稳步推进。走出去步伐加快，中山企业投资目的地扩至全球36个国家和地区。口岸通关环境不断优化，增设中山港海关，“单一窗口”累计申报业务量居全省前列，口岸提效降费取得阶段性成果。深化社会信用体系建设，市公共信用信息管理系统获评全省首个国家级特色性平台网站。

（四）城市建设扎实推进。开展城镇开发边界划定工作。拓展城市发展空间，总部经济区石岐片区动工建设，开展翠亨科学城城市设计国际咨询。交通基础设施建设力度加大，深中通道全面动工，广中江高速、中开高速、东部外环高速、西环高速（含小榄支线）、香海高速、南沙港铁路等有序推进，提升改造中心城区23条主干道路；阜民路阳光大道段主线、新沙水公路南头至东凤段、马石公路改造工程五桂山段、黄圃快线岭栏路至南三公路段、长江路主线南段、康华路、景观路、齐学路实现通车；中山港新客运码头加快建设。完成“三旧”改造项目84个，用地167.8万平方米，处置闲置土地928万平方米。旧楼加装电梯工作稳步推进。岐澳古道对市民开放。创建省公交示范市取得新进展，新开通、优化调整公交线路40条。海绵城市和地下综合管廊规划建设扎实推进。

（五）生态环境持续改善。认真落实中央环保督察“回头看”，督察组交办案件全部办结，顺利保障省环保督察组进驻督察。实施污染防治攻坚战三年行动计划，扎实推进15个流域水环境治理，整治内河涌98公里；高污染燃料禁燃区扩展至全市域，建成100个大气网格微观站，空气质量达标率为85.5%，空气质量改善幅度排全省第一；完成农用地土壤污染状况调查。农业面源污染防治不断强化，农作物绿色防控覆盖率达73.5%。划定生态保护红线。实施生态环境损害赔偿制度，实现重要区域生态补偿全覆盖。成功创建国家森林城市，9个镇获评“广东省森林小镇”，数量全省最多。建成三角金鲤湾和鲤鱼山、沙溪滨河（东段）、民众长堤等4个湿地公园。三乡镇成为全省唯一荣获2018中国最美村镇生态宜居奖的村镇。生态环保设施不断完善，中心和北部组团垃圾处理基地扩容工程持续推进，3家生活污水处理厂完成提标改造。推广新能源汽车2000多辆，建成充电桩395个。

（六）民生事业不断发展。基本完成十件民生实事。全市教育、社保、医疗、就业、住房保障等涉及民生的支出共计133亿元，增长12.1%。居民人均可支配收入增长7.6%。就业创业形势稳定，城镇新增就业6.2万人，促进创业9513人，城镇登记失业率为2.3%。教育现代化水平不断提升，全市新建改建扩建公办中小学40所，新增公益普惠幼儿园18所，新增幼儿园学位6135个，提供积分入学指标23805个，高考各批次录取人数和录取率再创新高。医疗卫生事业全面发展，二甲以上医院达20家，基本公共卫生服务项目增至14项，所有镇区全部建成中医馆，市博爱医院妇产科大楼基本建成。公共文化服务体系不断完善，完成中山纪念图书馆主体工程，成立香山粤剧研究院，实现行政村（社区）综合性文化服务中心全覆盖。体育事业蓬勃发展，在第十五届省运会获得金牌数排名全省第五，成功举办2018中山马拉松赛，建成社区体育公园15个。社会保障体系不断完善，社会保险参保963万人次，退休人员基本养老金提高5%，低保标准提高8.9%；率先在全省建立大病困难帮扶中心，特殊群体重特大疾病医疗救助比例超过90%，市级临时救助金支出增加247%，提高老人津贴、孤儿生活费、残疾人补贴标准。食品药品安全状况持续向好，食品抽检总体合格率达97.9%，居民食品安全满意度不断提升。

（七）社会建设不断加强。平安中山建设深入推进，“扫黑除恶”专项斗争扎实开展，打掉涉黑恶犯罪团伙291个；“智慧公安”项目荣获“2018年度中国十大社会治理创新奖”；立体化治安防控体系加快构建，全市刑事案件立案数下降4.1%。法治建设稳步推进，全面推广诉前联调和律师调解试点经验，所有镇区通过省法治镇（乡）创建活动验收。基层社会治理持续加强，大力开展社工服务改革，实施875个“博爱100”全民公益项目，在社区延伸建立37个全民公益园服务站点。养老服务加快发展，创新推行“1+2+N”社区居家养老服务，建立居家养老服务中心85个。科学应对极端天气灾害影响，有效抵御“山竹”等强台风侵袭。脱贫攻坚工作进展顺利，对口帮扶潮州市、肇庆市，对口帮扶云南昭通市扶贫协作，对口支援西藏工布江达县、新疆生产建设兵团第三师图木舒克市、四川甘孜州，以及与黑龙江佳木斯市对口合作等工作有序开展。对外人文交流活跃，国际朋友圈不断扩大。外事、港澳事务、台务、侨务、民族宗教、统计、审计、国防建设、优抚双拥、妇女儿童、红十字、老龄、残疾人、气象、人防、档案、地方志等工作取得新进步。

（八）政府建设取得进步。坚持以政治建设为统领，深入学习贯彻习近平总书记对广东重要讲话和重要指示批示精神，认真开展“大学习、深调研、真落实”工作，促进全市政府系统树牢“四个意识”，坚定“四个自信”，坚决做到“两个维护”。稳步推进法治政府建设，自觉接受人大监督，支持政协履行政治协商、民主监督、参政议政职能，认真做好建议提案办理工作，办复人大代表建议213件、政协提案303件，满意和基本满意率达100%。政务公开不断深化，行政复议等工作有序开展，矛盾化解取得新成效。加强廉洁政府建设，认真落实政府系统党风廉政建设主体责任，严格落实中央八项规定及其实施细则精神，强化审计等监督机制。加强政府绩效管理，切实改进作风，推动全市政府系统集中精力谋大事、抓大事，政务服务水平不断提高。

各位代表！过去一年成绩来之不易，这是以习近平同志为核心的党中央坚强领导的结果，是习近平新时代中国特色社会主义思想科学指引的结果，是省委省政府和市委正确领导的结果，是市人大及其常委会和市政协监督支持的结果，是历届政府接力耕耘的结果，是全市干部群众凝心聚力、团结奋斗的结果，是海内外各界关心帮助的结果。在此，我代表市人民政府，向全市人民，向各位人大代表、政协委员，向各民主党派、各人民团体和社会各界人士，向驻我市中央有关单位、人民解放军、武警官兵，向所有关心支持中山发展的港澳台同胞、海外侨胞和国内外友人，表示衷心的感谢！

回顾过去，是为了更好地展望未来。在看到成绩的同时，我们也清醒地看到，工作中仍然存在一些突出问题和薄弱环节，主要有：一是稳增长压力加大。重大战略发展平台缺乏，市场信心有待提振，产业投资乏力，发展后劲不足。二是经济高质量发展水平不高。创新对经济发展的支撑不够，高端人才和研发团队不足，重大创新平台、高水平大学缺乏，企业自主创新能力不强，传统产业转型升级仍处于爬坡越坎阶段，新动能未能真正挑起大梁。三是城市吸引力和影响力不够。城市面貌有待改善，城市形象有待提升，城市功能有待优化，对高端要素的吸引力和承载力不强。城市规划不到位、执行刚性不强，低效用地等问题突出，城市管理精细化水平有待提高。四是体制机制束缚有待突破。城市发展分散化、碎片化较为严重，资源要素统筹不够，体制机制改革有待深化，镇区发展活力有所弱化。五是污染防治任务艰巨。水环境尚未得到明显改善，大气治理任重道远，土壤、固废污染治理能力有待加强。六是公共服务还有短板。教育、医疗、出行等方面，与群众期待还有不小差距。七是营商环境有待优化。对比先进地区，我市在市场准入、投资贸易便利度、政务服务效率、营商成本等方面还有差距。八是干部队伍建设仍需加强。有些公职人员怕担当、不作为，开拓创新、攻城拔寨的精气神不足，有的视野和格局不够开阔。对于这些问题，我们将勇于面对、积极应对，不逃避、不退缩、不懈怠，认真筹划，大胆出招，果断破解，努力让中山在“二次创业”中重拾信心、再创辉煌。

2019年工作安排

各位代表！今年是新中国成立70周年，也是全面建成小康社会关键之年，做好今年的工作意义重大。习近平总书记对广东重要讲话和重要指示批示，为我们的工作提供了科学指引和行动指南。习近平总书记在庆祝改革开放40周年大会上的重要讲话，发出了新时代改革开放动员令，奏响了新时代改革开放再出发的最强音，为我们高举新时代改革开放旗帜、重整行装再出发，提供了根本遵循、注入了强大动力。站在新的历史起点上，我们要牢记总书记的亲切关怀和殷殷重托，进一步传承老一辈改革开创者的优良传统，大力弘扬敢闯敢干、敢为人先的改革精神，焕发改革开放再出发的精神状态和奋斗姿态，坚定沿着总书记指引的方向阔步前进，再创中山改革发展新局面。

根据市委工作部署，今年政府工作的总体要求是：高举习近平新时代中国特色社会主义思想伟大旗帜，全面贯彻党的十九大和十九届二中、三中全会以及中央经济工作会议精神，全面贯彻落实习近平总书记对广东重要讲话和重要指示批示精神，按照省委十二届历次全会和市委十四届历次全会部署，统筹推进“五位一体”总体布局和协调推进“四个全面”战略布局，坚持稳中求进工作总基调，坚持新发展理念，坚持推动高质量发展，坚持以供给侧结构性改革为主线，坚持深化市场化改革、扩大高水平开放，扭住建设粤港澳大湾区这个“纲”，统筹做好稳增长、促改革、调结构、惠民生、防风险、保稳定各项工作，进一步稳就业、稳金融、稳外贸、稳外资、稳投资、稳预期，提振市场信心，坚决打好三大攻坚战，着力提高发展平衡性和协调性，坚持不懈推进全面从严治党，保持经济持续健康发展和社会大局稳定，奋力把中山建设成为珠江东西两岸融合发展支撑点、沿海经济带枢纽城市、粤港澳大湾区重要一极，为广东实现“四个走在全国前列”、当好“两个重要窗口”贡献中山力量，以优异成绩庆祝中华人民共和国成立70周年。

2019年全市经济社会发展的主要预期目标是：地区生产总值增长6.5%左右，规上工业增加值增长6%，固定资产投资增长8%，社会消费品零售总额增长5%，一般公共预算收入增长5%，居民消费价格涨幅控制在3%以内，城乡居民人均可支配收入增长与经济增长基本同步，主要污染物总量减排和大气、水环境质量完成省下达的目标任务。

综观国内外形势，机遇和挑战相互交织。我国发展面临的环境更复杂，风险挑战更多，但仍处于并将长期处于重要战略机遇期，经济长期向好态势不会改变。现在已经进入“湾区时代”，我市处于粤港澳大湾区腹地，是大湾区重要节点城市，也是省委省政府布局的珠三角核心区与沿海经济带的交汇之地，将迎来新的更大战略机遇，但也面临更大挑战。只要我们深刻把握、全面用好全国全省发展的重要战略机遇期，坚持辩证思维和底线思维，坚持“五头并进”工作方法，深化改革开放，勇于创新突破，瞄准高质量发展要求，全市人民团结一致向前看，正视问题、勇于担当，就一定能战胜各种艰难险阻、风险挑战，保持经济持续健康发展和社会大局稳定。

新的一年，我们将着重做好以下工作。

一、抢抓机遇、高位谋划，全力推进粤港澳大湾区建设

抓住历史性机遇，贯彻落实好中央和省委的统一部署，谋划好关系中山长远发展的战略安排，以改革创新的精神高水平打造国际一流湾区城市。

积极推动环湾布局向东发展。顺应湾区城市发展的普遍规律，推动中山环湾布局，加快城市发展重心向东延伸。坚持将东部沿海片区与中心城区一体规划建设，从市级层面通盘考虑东部沿海片区功能定位和产业布局，努力打造国际一流、湾区顶尖的现代化滨海新城。加强中心城区与东部沿海片区互联互通，打通向东的交通联系，谋划推进博爱路、中山路、康华路向火炬区、翠亨新区腹地延伸，谋划孙文东路快速化改造。完善马鞍岛城市设计、片区规划、基础设施建设，着力建设现代化国际化创新型城市新中心，力争把马鞍岛打造成为新时代代表中山参与大湾区分工和建设的战略平台。加快对接南沙、前海、横琴自贸片区，争取创建成为自贸区新片区。

加快基础设施互联互通。充分发挥中山区位优势，系统研究全市交通基础设施建设，大力畅通对外综合运输通道，打造大湾区西部重要综合交通枢纽。大力发展轨道交通，推动深茂铁路深江段动工建设，加快南沙港铁路建设，尽快启动中南虎城际、广中珠澳高铁前期工作，争取深南高铁布局中山，推进广州地铁18号线延伸至中山，完善中山市域轨道交通规划。加快高速公路建设，推进深中通道、广中江高速、中开高速、西环高速（含小榄支线）、东部外环高速、香海高速建设，做好南中特大桥、西部沿海高速申堂互通、广澳高速世纪大道出入口工程、广东滨海旅游公路（中山段）前期工作。加快推进与大湾区各市在客货运、信息基础设施、能源、水利等方面合作。

加强市级统筹和推动镇区同向发力。坚持“四轮驱动”，以市镇政府机构改革为契机，优化市级政府机构职能，加强对规划、土地、交通、产业、平台、创新、生态等统筹力度，强化各组团的资源整合，破解资源利用“碎片化”、内部恶性竞争等问题，建立健全有效的工作机制，充分发挥各镇区工作积极性，实现城市功能合理布局、设施共建共享、综合效益最大化。发挥财政作用，保障镇区运行。

以广深港澳高层次合作为基础打造国际合作创新区。落实与广州的战略合作，有序推进两市重大规划衔接、基础设施对接等各项工作。加强与深圳对接，在科技创新、交通建设等领域，谋划推进一批重点合作事项。加快推进粤澳全面合作示范区建设。对接广深港澳科技创新走廊，大力引进港澳创新人才和团队，加强与广深港澳高校和科研机构合作，推动科研成果在中山转化应用。强化与港澳科技创新创业交流，在翠亨新区谋划建设粤港澳青年创新创业平台，吸引青年人才来我市创新创业。加强与国内外知名高校合作，争取启动与澳门科技大学合作的香山大学建设，积极筹备省市共建中山科技大学。积极创建粤港澳大湾区生物医药国际合作创新区。加强与国内外知名医疗机构合作，推动在中山设立高端医疗机构，争创区域医疗中心。坚持世界眼光和国际标准，大力优化创新创业环境，吸引全球产业、资本、技术、人才等高端创新要素集聚，使中山成为有梦想的创业者、创新者、投资者的理想向往之地。

二、坚持先进制造业和现代服务业双轮驱动，加快构建现代产业体系

把发展的着力点放在制造业上，强化现代服务业对实体经济的支撑，引导金融活水更好浇灌实体经济，发挥人才对高质量发展的引领作用，加快构建以创新为动力、实体经济为主体、现代服务业为支撑的现代产业体系。

增强科技创新能力。坚持创新是第一动力，充分发挥创新对经济发展的引领和支撑作用。实施高新技术企业“树标提质”行动，助推科技型企业发展壮大。鼓励企业加大研发投入、设立研发机构，培育发展一批工程实验室、工程技术研究中心、企业技术中心。继续推进中国科学院药物创新研究院中山研究院（华南分院）、哈工大无人装备与人工智能创新中心、复旦大学创新中心、香港大学生物医药技术国家重点实验室—广东药科大学分中心等重大创新平台建设，争取国家重大科技基础设施、省级以上重大创新平台布局中山，支持知名高校院所在我市设立科研机构。鼓励市内高校开展基础研究和应用基础研究。支持有实力的企业承担国家和省级重大科技项目。发展科技服务业和技术交易市场。促进科技金融产业融合发展，发挥创新创业投资引导基金作用，大力发展普惠性科技金融。加强知识产权保护，完善知识产权快速维权机制。

推动制造业高质量发展。瞄准世界科技前沿，大力发展健康医药、新一代信息技术、高端装备制造等战略性新兴产业，高起点建设先进制造业集群。推进市级产业平台建设，着力引进大型优质项目，支持上市公司、龙头企业总部建设。完善服务企业全生命周期公共技术服务平台体系，重点建设工业互联网、“互联网+”共享设计、智能制造、共性工厂等平台。探索建立企业高质量发展综合评价体系，重点评估企业用地、用工、用能等资源要素产出效率，逐步构建差别化配置机制，促进生产要素向高效益、高产出的区域和企业集聚。开展质量提升行动，推进国家电梯监督检验中心（广东）电梯安全风险评估基地、VDE鉴衡综合检测基地等质量平台建设，全面提升产品、工程、服务和环境质量。做好第四次全国经济普查工作。

大力发展现代服务业。完善现代服务业政策体系，推动生产性服务业向专业化、价值链高端延伸，推动生活性服务业向便利化、精细化、品质化发展。推进国家服务业综合改革试点，以金融、旅游、会展为突破口，推动服务业大发展。大力发展全域旅游，打造有特色、有影响力的大型旅游项目，加快推动重大文旅综合体项目落地，培育壮大文化旅游、乡村旅游等新兴旅游业态。统筹整合会展资源，规划建设专业化功能性展览馆，打造有国际影响力的特色会展品牌。实施重点产业培育工程，推进现代服务业集聚区、服务业示范园区建设。推动现代服务业与制造业融合发展，着力发展检验检测、研发创新、新兴信息技术服务、现代金融、高端商务商贸、现代物流等生产性服务业。

做强做优传统产业。通过“互联网+”、物联网、技术创新等手段，改造提升传统产业，引导传统产业以市场需求为导向，加快技术改造和产品创新，提高产品市场竞争力；以信息化建设为抓手，提升企业管理水平和市场运营能力；打造公共技术服务平台，为企业创新创造提供支撑；依托传统优势产业集群，通过“强龙头、搭平台、优服务”，努力将其打造成为企业总部所在地、技术创新地和市场运营地。

强化现代金融支撑。培育发展特色金融服务业，大力发展银行、证券、保险等金融机构。积极发展风险投资和创业投资。着力培育和引进上市企业，引导上市企业聚焦实体经济规范发展，支持企业改制上市、新三板挂牌，加快形成一批上市企业梯队，与省股权交易中心共建区域股权市场特色板块。支持本土金融机构做大做强。大力发展普惠金融，用好“助保贷”“过桥贷”、技改设备融资租赁等资金，加大对民营企业、小微企业支持力度。发挥纾困基金作用，支持企业解决股权质押风险。做好金融风险防控工作。

发挥投资关键作用。今年安排213个省市重点建设项目，总投资4136.2亿元，年度计划投资506.9亿元。完善项目招引全过程跟踪服务机制，及时协调解决项目推进过程中遇到的问题，全力推动项目落地投产。发挥基础设施项目所具有的民生和投资双重作用，加大基础设施投资力度。落实首台套等惠企政策，扩大工业投资规模。提高招商引资国际化水平，着力引进一批大型企业和重点产业项目。

建设高素质人才队伍。实行更积极、更开放、更有效的人才政策，创造更具吸引力的引才生态环境。坚持用好的平台留人，建设好大学、科研院所、院士工作站、博士后工作站、研究生联合培养基地等平台。大力引进和培养高水平创新创业科研团队，着力引进紧缺适用的特殊人才，加强企业技能人才队伍建设。实施青年优才工程，制定“雏鹰归巢”引才计划，吸引更多本地学子回中山创新创业。加强人才创新创业服务，大力发展人力资源服务业。研究制定“柔性引才”办法，推广“创新人才+创新项目”引才模式。加大人才资源整合力度，探索建立人才集团。办好中山人才节。激发人才干事创业潜力，用活用好存量人才，营造爱国奉献的良好氛围。

三、坚持深化市场化改革与扩大高水平开放两手抓，加快形成全面开放新格局

大力弘扬敢闯敢干、敢为人先的改革精神，重燃“逢山开路、遇水架桥”的干事激情，营造“鼓励创新、宽容失败、拒绝平庸”的社会氛围，注重改革系统性、整体性、协同性，把对外开放的大门越开越大，将改革开放进行到底。

深化重点领域改革。抓住牵一发而动全身的基础性、关键性改革，加快体制机制创新，减少体制机制对市场的束缚。深化“放管服”改革，接好用好省级下放的管理权限。完善市镇财政管理制度，深化预算管理，全面实施绩效管理。完善农村集体产权制度改革。加大国企改革力度，建立健全现代企业制度，做强做优做大市属国有企业。深化医药卫生体制综合改革，加快建立现代医院管理制度，全面实施公立医院薪酬制度改革，落实三级医院总会计师制度。

推进高水平开放。落实国家和省稳外贸、稳外资的政策措施，帮助企业开拓东盟、中东欧、非洲等新兴市场。积极参与“一带一路”建设，利用商会协会、侨务、中介服务机构等渠道，帮助企业走出去、引进来。加快培育发展外贸新业态，推进利和灯博中心市场采购贸易方式试点工作，争创跨境电商综合试验区。发挥信用保险的作用，扩大政策性出口信用保险覆盖面。鼓励先进技术、关键设备和零部件进口，扩大优质消费品进口。优化通关环境，推动口岸提效降费。建设境外展示展销中心、特色产业园区，促进经贸文化交流。

大力优化营商环境。坚决破除各种壁垒，营造国际化法治化市场化的营商环境。贯彻国家和省放宽市场准入政策，全面实施市场准入负面清单管理。推行公平竞争审查制度，清理和取消国有企业、民营企业、外资企业在资质资格获取、招投标、权益保护等方面差别化政策。推进审批服务“四办”改革，实现高频事项“最多跑一次”。加快“数字政府”建设，推进智慧政务平台建设，开展企业开办便利度评估，提高开办企业便利度。加快政务服务综合窗口建设，实现70%以上事项“一窗”分类受理。深化商事制度改革，推进商事登记全程电子化智能审批。优化提升市场监管效能，维护良好市场秩序。推进诚信体系建设。

激发各类市场主体活力。实施更有力度、更有温度的惠企、减税、降费政策，帮助企业轻装上阵、发展壮大。落实民营经济帮扶政策，支持民营企业做强做优。实施龙头骨干企业培育计划，壮大一批成长性高、带动性强的行业龙头企业和小巨人企业，推动小微企业上规模、中型企业上档次。加强对涉美加征关税清单企业的跟踪服务。大力弘扬企业家精神，依法依规保护企业家人身和财产安全。

四、坚持规划、建设、管理同向推进，着力提高城市品质和能级

坚持规划先行与建管并重相结合，突出问题导向，提高规划质量，强化执行刚性，着力破解用地突出问题，提高城市、建筑和景观设计水平，塑造特色风貌，营造宜居环境，创新管理服务，让中山更生态、更人文、更有韵味。

坚持规划引领城市发展。以国土、规划职能整合为契机，启动编制2035国土空间规划，统筹全域生态、生产、生活空间。高起点完善全域各功能区规划，加快优化市级产业平台规划和建设，推动各区域差异化、高质量发展。加强自然空间管控，严守生态控制线、城镇开发边界等刚性管控边界。优化规划管理体系，高标准配置公共服务设施，强化规划刚性约束，加强对各功能片区规划调整的统筹和指导。实施城市建设品质提升计划，加强建筑风格、建筑强度、建筑高度、城市色彩、第五立面、城市天际线等方面管控，改善城市总体风貌。推进特色小镇建设。

加强交通基础设施建设。大力推进交通强市建设，发展综合交通，构建高质量立体化交通体系，增强交通项目规划建设管理的系统性和协同性。加快快速干线建设，按照全市一盘棋、统一规划、同步实施的原则，推进主要通道快速化改造，完成长江路改造并全线通车，加快东二环、西二环、北二环、民众快线、黄圃快线、南朗快线、坦洲快线、岭栏路等干线公路建设，启动大涌快线、古镇快线、坦神北路、坦洲大道、105国道南线等干线公路建设，开展民古路小榄水道特大桥前期工作。谋划打通中心城区与高速出入口、枢纽站场之间的快速通道联系。加快市政道路改造，完成松苑路等23条道路改造，启动富华道、悦来南路等主干道路全要素提升改造，推进兴中道美化亮化并深入研究快速化改造的可行性。加强港口建设，加快新客运码头建设，加强港口智能化建设，完善中山港马鞍港区配套设施。完成省公交示范市验收。

大力推进集约节约高效用地。提高依法用地意识，开展国土和规划领域专项治理。加强土地资源统筹，优先保障公共基础设施和公共服务设施建设用地需求，最大限度地保障产业发展需求。提高单位土地产出效益，产业用地优先向战略性新兴产业、重大产业平台倾斜。用好用活“三旧”改造政策，加快批而未供和闲置土地处置。加快低效、低端、低质工业园区（厂房）改造，打造一批优质产业园区。划定工业控制线，推动镇村工业园区改造升级。结合第三次全国国土调查，摸清国土资源家底。

推动房地产市场平稳健康有序发展。坚持“房子是用来住的、不是用来炒的”定位，加强宏观调控和政策引导，加强房地产违法违规行为整治，严厉打击房地产领域的欺诈行为，改变房地产市场无序散乱状态，加大问题楼盘处置力度。坚持质量为上、安全为先，建立可管理、可追溯的房地产诚信体系，实施房地产市场主体信用记录和激励惩戒制度。

加快城市建设和更新步伐。实施“强心”战略，优化提升中心城区功能，统筹推进总部经济区布局和建设。找准城市更新的突破点，大力推进旧城连片改造，成熟一个推动一个，实现老城区新活力。坚持用“绣花”功夫，推进老城区城市修补、生态修复，挖掘古村落、古建筑等人文资源，加强名镇名村、历史文化街区和城市特色风貌管理，继续推进岐澳古道活化利用。开展老旧小区改建综合服务中心试点，鼓励老旧住宅楼加装电梯，推进老旧小区管道燃气入户工作，研究中心城区综合停车场建设。加快翠亨新区基础设施建设，推进海绵型试点项目和地下综合管廊建设。推进智能电网规划建设，建设220千伏君兰、团结、古海输变电工程。推进“厕所革命”，建设和集中改造一批公厕。加快城区垃圾转运站改造。

加强城市智能化、科学化、精准化管理。整合优化城市管理职能，构建大城管格局。加快数字城管建设，推进交通、市政、安防、公共服务和市民生活各领域的智能化应用。开展交通文明畅通提升行动，加快“智慧交通”建设，优化城区交通信号灯系统，整治交通违规违章行为，提高道路通行能力。促进城区中小道路交通“微循环”，加强道路建设交通仿真评估，缓解因道路施工引起的交通拥堵。加强网约车、共享单车、电动单车管理。提高供水、供气、供电等服务保障能力。规范户外广告。

五、坚持美村、兴业、富民三管齐下，大力推动乡村振兴

坚持城乡互动、共同发展，推动城乡基础设施互联互通、公共服务普惠共享、资源要素平等交换、生产要素充分对接，带动乡村产业、人才、文化、生态和组织振兴，让农业更优、农村更美、集体更强、农民更富。

加强对农村集体经济组织的管理和指导。加大农村集体经济扶持力度，建立健全支持农村集体经济发展的政策体系和管理制度，强化集体经济组织发展经济和服务农村的职能。引导农村干部深化改革、解放思想、拓宽思路，结合老旧厂房改造和资源整合，探索混合经营等多种形式，推动资源变资产、资金变股金、农民变股东，助推农村集体经济转型升级。

推进农业现代化。实施特色优势产业提质增效工程，大力发展优质水产、精品花木、特色水果、放心蔬菜等特色产业。推进现代农业产业园建设，打造农业发展新载体。扶持发展“菜篮子”工程，增加产品有效供给。推进农业经营主体增量提质，建设一批示范性家庭农场和示范性农民合作社。开展耕地质量保护与提升行动，推进高标准农田建设，提升农业综合生产能力。做好粮食安全工作。实施质量兴农、品牌兴农行动，创建农业标准化示范基地，建设农产品质量安全追溯体系，加强动植物疫病防控。强化绿色兴农，鼓励开展生态养殖。大力推进科技兴农，提高农业科技创新能力。推进“大棚房”问题清理整治。拓展农业功能，推进农业与旅游、教育、文化、健康养老等产业融合发展。

推进生态宜居美丽乡村建设。深入实施农村人居环境综合整治，持续开展“三清理”“三拆除”“三整治”行动，加强村居水环境和生活垃圾治理，推进农村污水处理设施建设，大力整治脏乱差现象，改善村容村貌。加强乡村生态保护与修复，推进乡村绿化美化。大力发展乡村旅游，推进乡村旅游公路建设，创建一批美丽宜居村和特色精品村。深入实施“粤菜师傅”工程。制定乡村可开发文物建筑资源和旅游开发资源目录，加强古村落、古民居、古建筑等人文资源保护和开发。完善镇村公共文化服务体系，管好用好镇村公共文化设施。加强乡村治理，发挥村（社区）法律顾问的作用，完善农村法治建设机制。

保障和改善农村民生。加强农民就业创业服务，建立镇村助农服务中心，拓展农民增收渠道，促进农民增收致富。改善镇村薄弱学校办学条件。完善镇村交通网络，加强镇村信息化、物流、电力、燃气等基础设施建设。推进社区卫生服务机构规范化、标准化建设，改善基层医疗卫生服务条件。加强农村干部培训，推动农村专业人才队伍建设。

做好精准扶贫、精准脱贫工作。坚持扶贫与扶智、扶志相结合，加强对全市相对贫困村、贫困人口帮扶。做好潮州市、肇庆市对口帮扶工作。继续做好对口帮扶云南昭通市扶贫协作，对口支援西藏工布江达县、新疆生产建设兵团第三师图木舒克市、四川甘孜州，以及与黑龙江佳木斯市对口合作等工作。

六、坚持生态保护与环境治理相促进，建设美丽中山

坚定践行绿水青山就是金山银山的理念，实行最严格的生态环境保护制度，把生态保护放在优先位置，打好碧水、蓝天、净土三大战役，让百姓对环境质量改善的获得感越来越强。

加强水环境治理。把水污染治理放在重中之重位置，落实河长制、湖长制，坚决打好碧水攻坚战。强化饮用水源地保护，完成饮用水源保护区“划、立、治”任务，守护好生命之水。坚持系统治水理念，加快水质自动监测平台建设，全面推进15个流域水环境治理工程项目开工建设，抓好中心组团157条河涌325公里黑臭水体整治。坚持岸上截污、河底清淤、接通水体相结合，加快推进黑臭水体综合整治，完善污水管网建设。加强近岸海域污染防治。加强畜禽养殖污染治理，强化农业面源污染防治。加大水利设施建设力度。

加强大气、土壤、固废污染防治。加强工业污染、移动源污染防治，推进挥发性有机物综合整治，加强建筑工地扬尘防控管理，降低污染物排放。增设200个大气微观站，提升天气预警管控能力及响应速度。加强土壤污染防治，完成重点行业企业用地土壤污染状况调查，建立污染地块优先管控名单，推进污染地块整治和修复。大力推行垃圾分类，提升固体废物处置能力，加快危险废物综合处理项目建设，实现中心和北部组团垃圾处理基地扩容工程投入使用。加大执法力度，严厉打击环境违法行为。做好中央环保督察“回头看”和省环保督察反馈意见整改工作。

营造更多绿色空间。建设高品质国家森林城市，推进翠亨国家湿地公园、中山国家森林公园、国家级自然保护区三块“国字号”金字招牌建设。加快凤凰山、铁炉山、丫髻山等森林公园建设，推进金斗湾、崖口、竹排等省市级生态湿地公园建设，推进金钟湖公园群等城市公园建设。推进四个组团儿童公园建设。开展绿化美化提升行动，支持镇区建设森林小镇。加强海岸线保护与利用。做好森林防火管控，严格规范林地使用管理。

推进绿色低碳循环发展。引导企业节能降耗，推动企业开展清洁生产。提高天然气等清洁能源应用水平，壮大风电等清洁能源产业。加快推进国家低碳城市试点建设，创新建设近零碳排放示范工程。积极发展绿色交通，大力推进使用新能源公交车，加快充电基础设施建设。发展装配式建筑。倡导绿色低碳生产生活方式。

七、坚持保障和改善民生与加强社会治理并重，切实增进人民群众福祉

坚持以人民为中心的发展思想，时刻把百姓的安危冷暖放在心上，着力解决好群众反映强烈的痛点堵点难点问题，把社会治理的底板加固、短板补齐，使社会既生机勃勃又井然有序，努力让改革发展成果更多更公平地惠及人民。

优先发展教育事业。构建科学完善的教育体系，提高学前教育、高等教育、民办教育质量。大力推动公办幼儿园和民办普惠幼儿园建设，年内新增幼儿园规范化学位5000个。推进集团化、学区化办学，实施新一轮初中振兴计划和普高提质计划，新建改建扩建公办中小学28所。大力发展职业教育，调整中职教育布局和专业布局，构建中职—高职—应用型本科衔接的职业教育体系。加快推进高水平大学建设，加强市内高校重点学科建设，提升高等教育发展水平。推进民办学校分类管理，鼓励社会力量举办高质量的民办学校和国际学校。实施学生体质提升行动，加强学生体育锻炼，开展“爱眼护齿”工程，提高学生健康水平。深化教师队伍建设改革，启动市级教师发展中心建设，创建国家教师教育创新实验区。

提高就业创业质量。坚持就业优先战略和积极就业政策，实现城镇新增就业4.5万人，城镇登记失业率控制在3%以内。深入落实进一步促进就业若干政策措施，促进扩大高校毕业生等青年群体、就业困难人员、异地务工人员多渠道就业，确保就业局势总体稳定。鼓励创业带动就业。推行终身职业技能培训，提高劳动者技能水平。

加强社会保障建设。配合实施养老保险省级统筹，扩大城镇职工养老保险覆盖范围。落实职业年金制度。完善基本医疗保险办法，推进跨省医保异地就医直接结算。加强“两定机构”执法检查，完善社保基金监督长效机制。加大兜底保障力度，完善低保认定条件，制定特困人员供养制度，完成低保低收入家庭危房改造。提升养老服务质量，完善社区居家养老服务体系，稳步扩大经济困难失能老人照护覆盖面。推进医疗机构与养老服务融合发展。加大慈善救助力度。

推进健康中山建设。深入实施健康城市三年行动计划。推进市人民医院新院区、市儿童医院建设，动工建设市第三人民医院改扩建工程，积水潭骨科医院开业运营，市博爱医院妇产科大楼、市第二人民医院南区分院扩建项目投入使用。大力推进高水平医院建设，争取入选广东省第三批“登峰计划”重点建设医院。推进分级诊疗和医联体建设。深化药品供应保障制度改革，实施公立医疗机构药品跨区域联合集中采购。提高公共卫生应急保障能力，做好各类传染病防控。推动“互联网+医疗健康”发展，改善群众就医体验。加大高层次和紧缺适用专业医疗人才培育和引进力度。提升老龄健康、妇幼健康和职业健康服务水平。

深入推进平安中山建设。防范化解重大社会风险，维护社会和谐稳定。持续纵深推进“扫黑除恶”专项斗争，严密防控涉黑拐枪爆恐、新型网络犯罪、盗抢骗、黄赌毒等违法犯罪活动。推动“智慧公安”建设成型发展，提高社会治理科学化、智能化、精准化水平。推广智慧项目应用，探索建设“无人车管所”，推进智慧安防进校园、自助办证进村居。推动跨区域警务协作，提高跨区域犯罪治理能力。加强政府债务管理，防范财政运行风险。完善从农田到餐桌的监管执法链条，强化企业主体责任，不断提高食品安全保障水平。统筹、整合、优化应急救援力量，加强道路交通安全、消防安全、生产安全、药品安全、校园安全、气象安全和公共安全整治，严防各类重特大事故发生。

加强和创新社会治理。广泛推动全民参与社会治理，推进“博爱100”全民公益行动和全民公益园建设。培育发展社区社会组织，深化社工服务改革，建立健全异地务工人员互助平台。完善积分入学、积分申请公租房等制度。发挥村（居）特别委员作用，全面开展村（居）务监督委员会考核。加强法治文化宣传，优化公共法律服务体系，促进基层治理法治化。完善社会矛盾纠纷多元化解机制，加强矛盾纠纷排查调处及重大政策社会稳定风险评估。做好戒毒、社区矫正、安置帮教工作。强化劳动保障监察执法，开展“治欠保支”专项整治，有效维护劳动者合法权益。

推进文化旅游繁荣发展。培育和践行社会主义核心价值观，大力弘扬以爱国主义为核心的民族精神和以改革创新为核心的时代精神。创建国家公共文化服务体系示范区，优化市镇村（社区）三级公共文化设施网络，引导社会力量参与公共文化服务。完善图书馆和文化馆总分馆体系，实现中山纪念图书馆投入使用。大力发展文化产业，做大做强游戏游艺、红木文化、灯饰文化等特色文化产业。加强优秀历史文化资源保护、传承和利用，促进文化旅游互动融合发展。开发和利用好孙中山文化资源，实施香山先贤文化资源活化利用工程，建设孙中山文化国际交流中心，提升孙中山故里旅游景区管理服务水平。用好用活孙文西路文化旅游街区、博物馆群、环铁城道等历史文化资源，修缮郑观应等名人故居。谋划推进科技馆、工业馆、美术馆等城市文体设施建设。研究建设华侨公园。繁荣文化艺术。创建国家全域旅游示范区，提升旅游服务品质。大力发展体育事业，深入开展全民健身活动，构建城市社区15分钟健身圈，实现新建社区体育设施覆盖率100%。

加强退役军人服务管理，做好优抚双拥工作。做好外事、港澳事务、台务、侨务、民族宗教、统计、审计、国防建设、妇女儿童、红十字、老龄、残疾人、气象、人防、档案、地方志等各项工作。

全力办好十件民生实事。一是停车信息服务平台建成投用；二是推进老旧小区加装电梯；三是智慧安防进校园；四是建成开放一批公园；五是增加幼儿园和公办中小学学位；六是“爱眼护齿”健康行动；七是优化政务服务；八是异地务工人员子女关爱行动；九是上线中山“阳光食品”APP；十是提升道路通行能力。

八、坚持提高治理能力和加强自身管理相得益彰，努力建设法治廉洁高效政府

坚持以习近平新时代中国特色社会主义思想为指导，全面落实新时代党的建设总要求，坚定不移推进全面从严治党，树牢“四个意识”，坚定“四个自信”，坚决做到“两个维护”，锻造忠诚干净担当的高素质干部队伍，努力建设人民满意的政府。

加强党的领导和党的建设。全面贯彻落实习近平总书记对广东重要讲话和重要指示批示精神，切实用以统一思想、指导实践、引领发展。旗帜鲜明讲政治、抓政治，始终在政治立场、政治方向、政治原则、政治道路上，同以习近平同志为核心的党中央保持高度一致。坚持把全面从严治党要求贯穿于政府工作全过程，全面推进政治建设、思想建设、组织建设、作风建设、纪律建设，把制度建设贯穿其中，深入推进反腐败斗争，推动政府系统党的建设规范化、标准化。加强政府系统意识形态安全建设，为改革发展稳定明确思想引领、汇聚强大力量、凝聚广泛共识。

建设法治政府。依法全面履行政府职能。加强关键领域政府立法，推进行政复议规范化，加强行政应诉工作。全面落实行政执法责任制，进一步强化行政执法监督。加大全民普法力度，拓宽公共法律服务的领域和深度。自觉接受市人大及其常委会的监督，积极支持市政协履行政治协商、民主监督、参政议政职能，认真办理人大代表建议和政协委员提案。持续深化政务公开，大力推进重点领域信息公开，及时回应社会关切。

建设廉洁政府。坚持把纪律和规矩挺在前面，把政府系统党风廉政建设引向深入。认真落实中央八项规定及其实施细则精神，自觉接受纪律监督、监察监督、派驻监督和巡视监督。坚决铲除滋生腐败的土壤，紧盯重大工程、重点领域、关键岗位，强化廉政监督和廉政风险防控。持续推进反腐倡廉，坚持无禁区、全覆盖、零容忍惩治腐败。加强作风建设，力戒形式主义、官僚主义。严控“三公”经费支出。发挥好审计监督作用，营造风清气正的良好政治生态。

建设高效政府。正确处理好政府与市场的关系，加快政府职能转变。构建一体化平台、大数据共享、部门协同联动的电子政务体系，提高政府治理能力。持续整治“文山会海”，进一步压减文件和会议数量。优化政府绩效管理，建立与高质量发展要求相适应的绩效评价体系。强化督查督办，不折不扣落实好中央和省委省政府、市委的部署。完善正向激励机制，营造崇尚实干、勇于担当的良好氛围。加强干部队伍建设，提高政治素质，培养专业能力，教育引导广大干部敢于斗争、善于斗争，打造高素质专业化公务员队伍。

各位代表！新时代改革开放再出发的号角已经吹响。让我们紧密团结在以习近平同志为核心的党中央周围，在省委省政府和市委的坚强领导下，不忘初心、牢记使命，凝心聚力、接续奋斗，以新时代新担当新作为，展现新气象、再创新局面，努力实现省委赋予的“三个定位”，奋力交出“四个走在全国前列”、当好“两个重要窗口”的优异中山答卷。
